# Gambling Disorder as a Behaviorally Driven Systemic Medical Condition: From Reward Circuitry to Multi-System Morbidity

**DOI:** 10.3390/clinpract16080137

**Published:** 2026-07-26

**Authors:** Martina Ballerio, Piercarlo Minoretti, Enzo Emanuele

**Affiliations:** 1 Center for Research in Medical Pharmacology, University of Insubria, I-21100 Varese, Italy; mballerio2@uninsubria.it; 2 Cantonal Psychiatric Clinic, Cantonal Sociopsychiatric Organization, 6850 Mendrisio, Switzerland; 3 Department of Social Sciences, Miguel de Cervantes European University (UEMC), 47012 Valladolid, Spain; scientific.direction@studiominoretti.it; 4 Studio Minoretti, I-23848 Oggiono, LC, Italy; 5 2E Science, I-27038 Robbio, PV, Italy

**Keywords:** gambling disorder, allostatic load, cardiovascular disease, metabolic syndrome, glucagon-like peptide-1 receptor agonists, behavioral addiction, systems medicine

## Abstract

Historically framed as a behavioral addiction, gambling disorder (GD) has had its somatic dimension neglected by standard clinical evaluation. This is a narrative, hypothesis-generating review focusing on the somatic aspects of GD. Cross-sectional and prospective evidence increasingly shows a somatic phenotype distributed across four axes: cardiovascular (hypertension, angina, stroke, acute stress-induced cardiac events), metabolic (obesity, type 2 diabetes, chronic liver disease), sleep-related (insomnia, poor sleep quality, daytime sleepiness), and—as an emerging and insufficiently characterized dimension—neurological (clustering with seizures and frontal lobe epilepsy). Three candidate biological systems may translate this exposure into multi-system physiology: (i) a hyperreactive mesostriatal reward circuit hypothesized to sustain a behavioral cluster of smoking, hazardous alcohol use, low physical activity, and unhealthy diet; (ii) an integrated stress response postulated to shift from acute hyperreactivity to chronic basal cortisol blunting and reduced vagal tone; and (iii) putative alterations in peripheral neurotrophic signaling and frontal-callosal structure, marked by elevated peripheral brain-derived neurotrophic factor and by frontal-callosal white-matter alterations—with cumulative allostatic load serving as a conceptual integrating frame. We propose that GD can be a behaviorally driven systemic medical condition, warranting internal medicine involvement alongside addiction psychiatry. Within this framework, glucagon-like peptide-1 receptor agonists emerge as an exploratory therapeutic hypothesis warranting dedicated evaluation in GD, given their action on the mesolimbic reward substrate and phase 3 evidence in adjacent cardiometabolic, hepatic, and sleep conditions.

## 1. Background

Gambling disorder (GD)—classified among the addictive disorders by the DSM-5 [1] and framed as a behavioral addiction within the ICD-11 nosological framework [2]—has been historically approached as a condition whose harm spectrum is limited to the psychiatric [3], financial [4] and social [5] aspects through which its diagnostic criteria are formulated. The conceptual architecture inherited from this framing has assigned the clinical care of the patient almost exclusively to addiction psychiatry [6] and to the structures of mental health [7], and has shaped the trajectory of research and treatment over the last two decades.

A growing body of survey-based, cohort, and case–control evidence has, however, increasingly indicated that the morbidity of the disorder is not exhausted by the domains that the original nosology was designed to capture. Specifically, the somatic conditions reported in patients with GD distribute across multiple organ systems, recur with a consistency that adjustment for the principal confounders does not attenuate, and admit a mechanistic reading in which a limited number of physiological pathways can translate the repeated behavioral exposure into a recognizable clinical profile. Taken together, this pattern of observations raises the question of whether GD should be conceptualized—at least in part—as a behaviorally driven systemic medical condition whose somatic burden is constitutive of the disorder rather than incidental. The reading proposed here follows the trajectory from the mesostriatal reward circuit [8] through the behavioral cluster it sustains to the multi-system somatic burden, with the integrated stress response [9] acting as the principal translational machinery and cumulative allostatic load [10] proposed as a conceptual integrating frame.

For the purposes of this hypothesis-generating review, we consider that a psychiatric or behavioral disorder fulfills the operational definition of a systemic medical condition when four criteria are jointly satisfied: (i) the somatic burden is distributed across multiple, apparently independent organ systems in a stratifiable and reproducible fashion, forming a somatic phenotype rather than a scattered set of comorbidities; (ii) the associations persist, at least in part, after adjustment for the principal confounders known to co-occur with the disorder, or in prospective designs; (iii) a mechanistic substrate connecting the behavioral exposure to the multi-organ morbidity can be plausibly articulated at the systems level, even if not fully demonstrated in humans; and (iv) the clinical implications extend beyond the disciplinary field in which the disorder was originally circumscribed, warranting the structured involvement of internal medicine alongside the primary specialty. The framework is proposed here as a conceptual, hypothesis-generating model rather than as an established nosological definition, and is intended to structure the current evidence base and to identify the empirical work required for its further validation. The mere co-occurrence of multiple medical comorbidities with a psychiatric diagnosis is not sufficient, since it does not require a shared mechanistic architecture and does not, by itself, warrant a re-classification of the disorder.

Our specific aims were (i) to present the population-level evidence on the somatic phenotype of GD across the cardiovascular, metabolic, sleep, and neurological dimensions, (ii) to articulate a systems-level pathophysiological framework that links the repeated behavioral exposure to the multi-organ profile through three overlapping, mechanistically coupled biological systems, and (iii) to outline the clinical, organizational, and therapeutic implications that may follow from approaching GD as a complex medical condition—with attention to the candidate role of glucagon-like peptide-1 receptor agonists (GLP-1 RAs) as a pharmacologic class whose mechanism of action and phase 3 evidence base intersect with the systems-level architecture of the disorder. In presenting the evidence, we distinguish throughout between epidemiological association, possible mediation, mechanistic plausibility, and demonstrated causality, with hedged language reserved for the levels of inference not yet supported by direct empirical evidence in GD.

## 2. Methods

For this narrative, non-systematic review, PubMed/MEDLINE, Embase, Scopus, and the Cochrane Library were searched from January 2000 to April 2026 (final search date), with retention of selected earlier publications where these remained pertinent to the conceptual argument. The population descriptors GD, pathological gambling, and problem gambling were cross-referenced with somatic domain descriptors covering somatic comorbidities, cardiovascular disease, hypertension, stroke, angina, takotsubo cardiomyopathy, metabolic syndrome, obesity, type 2 diabetes, chronic liver disease, sleep, insomnia, daytime sleepiness, neurological disease, seizures, epilepsy, frontal lobe epilepsy, hypothalamic–pituitary–adrenal axis, cortisol, dehydroepiandrosterone, heart rate variability, baroreflex, respiratory sinus arrhythmia, white-matter integrity, dopaminergic neurotransmission, allostatic load, and pharmacological treatment. Given the lack of direct GD-specific evidence, searches concerning GLP-1 RAs were extended to addiction, substance use disorder, alcohol use disorder, nicotine use disorder, and reward processing, and complemented by retrieval of the principal phase 3 trials of these agents in metabolic, cardiovascular, hepatic, and sleep disturbances. Reference lists of retrieved articles and pertinent reviews were screened to identify further sources. Eligible publications were conducted in human populations and reported in English, comprising population-based surveys, cohort studies, case–control and cross-sectional investigations, randomized controlled trials, mechanistic experiments, and neuroimaging studies, together with reviews used for contextual framing; preclinical animal evidence was additionally incorporated where directly relevant to the mechanistic argument on GLP-1 RAs. Suicidal behavior [11] and psychiatric comorbidities [12]—while constitutively related to the disorder [13]—were excluded from the analytical scope, as they represent outcomes whose conceptualization differs from the somatic phenotype proposed here. Conference abstracts that lacked accompanying full data and material that had not undergone peer review were also excluded. Consistent with our integrative, hypothesis-generating objective, studies were selected purposively rather than exhaustively, with prioritization based on methodological quality, sample size, representativeness of the source population, and direct relevance to the systems-level conceptualization of GD. The literature searches described above were executed iteratively over the drafting period, refined and expanded as the argument matured, and complemented by hand-searching of reference lists and by targeted retrieval of pertinent studies flagged during author discussion. Because the boundary between records identified, screened, and retained is not sharply defined in this narrative, iterative workflow, we do not report quantitative record-tracking figures, which would suggest a level of systematicity and adherence to a predefined protocol that the narrative architecture of this review does not embody. Consistent with the integrative, narrative nature of the review, a formal risk-of-bias appraisal was not undertaken. The rationale for this choice was threefold. First, the heterogeneity of study designs assembled here—cross-sectional and prospective epidemiological studies, small mechanistic investigations, diffusion-weighted MRI and PET studies, randomized controlled trials in adjacent indications, and preclinical animal models—is not amenable to a single validated appraisal instrument, since tools such as the Newcastle–Ottawa Scale, ROBINS-I, and Cochrane RoB 2 apply to distinct study designs and outcome types. Second, the objective of the review is to integrate converging lines of evidence across epidemiology, pathophysiology, and therapeutic plausibility, rather than to derive a pooled effect estimate for which the weight of individual study quality would be a central determinant. Third, the methodological limitations of the principal individual studies are discussed transparently on a study-by-study basis, so that the reader can appraise the strength of each observation within its own methodological context.

## 3. Epidemiological Evidence for the Somatic Phenotype of GD

The population-level evidence for the somatic phenotype of GD distributes across four dimensions—namely, cardiovascular, metabolic, sleep, and neurological. Each subsection reports the epidemiological associations documented in survey-based, cohort, and case–control studies. The evidence supporting the four dimensions is not equally developed: the cardiovascular, metabolic, and sleep dimensions rest on multiple population-based estimates and, in several cases, on prospective or case–control designs, whereas the neurological dimension rests on a smaller evidence base and is best regarded as an emerging and insufficiently characterized area within the somatic phenotype.

Of note, the exposure categories present in the reviewed literature span a graded continuum from participation to diagnosis, and are not clinically or methodologically equivalent. Participation-based measures (any past-year gambling, recreational gambling) capture the general population of people who gamble; screening-based measures (at-risk gambling, problem gambling, probable pathological gambling—as assessed, for instance, by the Lie/Bet questionnaire, the South Oaks Gambling Screen, or the Problem Gambling Severity Index) capture individuals with clinically significant gambling problems who may or may not meet formal diagnostic criteria; and diagnosis-based measures (pathological gambling by DSM-IV, gambling disorder by DSM-5 or ICD-11) capture the clinical population. In this review, associations reported from participation-based samples are read as useful background information on gambling exposure in the general population rather than as direct estimates of the association attributable to disordered gambling itself, since a participation-based sample includes a majority of gamblers who do not carry the disorder; only associations reported from screening- or diagnosis-based samples approximate the association attributable to the disorder itself. Convergence of associations across the three exposure levels strengthens the interpretation; divergence would restrict the inference to the diagnostic subset.

### 3.1. Cardiovascular Disease

Cardiovascular morbidity represents the most consistently reproducible somatic association of GD across the published literature. The largest body of evidence derives from cross-sectional, population-based surveys. In the 2001–2002 wave of the U.S. National Epidemiologic Survey on Alcohol and Related Conditions (NESARC), assessment of 43,093 adults aged 18 years and older identified lifetime pathological gambling—against the low-risk reference—as associated with diagnosed tachycardia (odds ratio [OR] 1.77, 95% confidence interval [CI] 1.05–2.97) and angina (OR 2.35, 95% CI 1.33–4.15), with both estimates persisting after adjustment for demographic characteristics, body mass index (BMI), alcohol use, nicotine dependence, and mood and anxiety disorders [14]. The older-adult NESARC subsample of 10,563 participants aged 60 years and older confirmed the association with angina, with past-year prevalence of 22.7% among lifetime disordered gamblers against 8.8% among adults without a history of regular gambling, after analogous adjustment [15]. More recent datasets have extended this profile to hypertension and cerebrovascular outcomes. The Prevention and Etiology of Gambling Addiction Study in the U.S. (PEGASUS), a Maryland-based prospective cohort that enrolled 1195 participants between 2015 and 2022 with deliberate oversampling of gamblers (34.2% of whom met study criteria for lifetime probable pathological gambling), identified high blood pressure and stroke among the strongest physical-health associations [16]. In a Swedish nationwide retrospective case–control study covering 2005–2019, 3592 patients with GD were matched one-to-two for age and sex with 7174 controls drawn from national registers; the prevalence of cardiovascular disease was 18% among patients with GD against 12% among controls, with women showing a higher prevalence of cardiovascular comorbidity than men [17]. In the 2023 California Health Interview Survey, any past-year gambling was associated with hypertension at an OR of 1.35 (95% CI 1.24–1.48) in a logistic-regression model adjusted for sex and age [18]; notably, exposure in this sample was measured as any past-year gambling rather than as a diagnostic category.

The longitudinal evidence—though more limited in scope—proved directionally concordant with the cross-sectional observations. In the older-adult NESARC analysis of 10,231 participants aged 55 years and older who completed both Wave 1 (2001–2002) and Wave 2 (2004–2005), at-risk, problem, and pathological gambling at Wave 1 predicted incident arteriosclerosis and incident heart disease at Wave 2, independently of baseline sociodemographic characteristics, psychiatric comorbidity, substance use, and BMI [19]. The prospective design and the adjustment set place this finding above the cross-sectional evidence in terms of temporal sequence, although the three-year inter-wave interval remained short relative to the natural history of atherosclerotic disease. A clinical observation that may further consolidate the cardiovascular association is takotsubo cardiomyopathy—a syndrome of reversible left ventricular dysfunction mediated by exaggerated sympathetic stimulation and triggered by acute emotional stress [20]. Gambling losses have been documented among the precipitating events in a prospective clinical series of stress cardiomyopathy [21], although a systematic estimate of the relative risk in this population has not been provided. The overall direction of the cardiovascular association is broadly consistent across the exposure continuum. However, direct comparisons of the size of the associations across the individual studies are not undertaken here, since the studies differ in exposure definition (any past-year gambling *versus* screening-based versus diagnosis-based measures), in outcome (hypertension *versus* any cardiovascular disease), in source population (state-level, national-registry, or clinical), and in statistical estimator (adjusted odds ratios *versus* unadjusted prevalence contrasts), so that direct magnitude comparisons across the estimates could be misleading.

### 3.2. Metabolic Disease

Metabolic morbidity constitutes the second clinical dimension along which GD shows a somatic signature, with consistent observations on obesity, type 2 diabetes, and excess chronic liver disease. The obesity association appears the most reproducible across study designs. In a community case–control study of 95 individuals with pathological gambling (South Oaks Gambling Screen score ≥ 5) and 91 controls recruited by random digit dialing, patients with GD showed a higher BMI than controls and a higher prevalence of obesity, with the number of medical conditions correlating with gambling severity [22]. In the Danish Health and Morbidity Surveys of 2005 and 2010, past-year problem gambling—a screening-based exposure category investigated by the Lie/Bet screening tool [23]—was associated with obesity at the population level, with the association persisting after adjustment for sex, age, education, cohabitation status, and other risk factors [24]. In a community sample of 207 young adults aged 18–29 years with subsyndromal GD, 10.6% were classified as obese (BMI ≥ 30 kg/m^2^) and 23.7% as overweight (BMI ≥ 25 kg/m^2^), and the obese subgroup reported greater monetary losses to gambling than the normal-weight subgroup—indicating that the obesity-GD association is detectable already in young problem gamblers below the diagnostic threshold [25]. In the Swedish nationwide case–control study cited above, the prevalence of obesity was 7% among the 3592 patients with GD against 3% among the 7174 matched controls [17]. Longitudinal data on body weight in GD remain limited but directionally concordant. In a one-year follow-up of 160 non-treatment-seeking young problem gamblers aged 18–29 years (102 normal-weight, 35 overweight, 23 obese at baseline), baseline obesity predicted smaller improvements in gambling symptom severity after one year, independently of nicotine use, sex, age, and baseline gambling severity [26]. This observation identifies obesity as a prognostic stratifier in young gamblers and supports a potential bidirectional reading of the obesity-GD relationship. Type 2 diabetes is another metabolic comorbidity with replicating evidence across two large datasets. In the Swedish nationwide case–control study, the prevalence of diabetes was 5% among patients with GD against 2% among matched controls [17]. In the 2023 California Health Interview Survey, any past-year gambling was associated with diabetes at an OR of 1.32 (95% CI 1.16–1.49) in a logistic-regression model adjusted for sex and age [18]. An indirect association with excess hepatic disease also emerged from the historical NESARC dataset, in which lifetime pathological gambling was associated with diagnosed cirrhosis (OR 3.90, 95% CI 1.11–13.72) and other liver disease (OR 2.98, 95% CI 1.07–8.26), with both associations persisting after adjustment for BMI, alcohol abuse and dependence, nicotine dependence, and mood and anxiety disorders [14]. The wide confidence intervals around the cirrhosis estimate reflected the relatively low number of affected cases, and prospective replication would consolidate the hepatic component of the metabolic phenotype. The overall direction of the metabolic association—across obesity, type 2 diabetes, and chronic liver disease—is likewise broadly consistent across the exposure continuum, with direct comparisons of the size of the associations across studies not undertaken here for the reasons detailed at the closing of Section 3.1.

### 3.3. Sleep Disturbances

Across population-based, community, and clinical samples, GD has shown consistent associations with insomnia symptoms, subjective sleep quality, and excessive daytime sleepiness. The population-based evidence provided the most informative estimates of prevalence. In the National Comorbidity Survey-Replication, 67.7% of adults with pathological gambling and 45.9% of those with problem gambling reported at least one sleep complaint, compared with the lower prevalence observed in respondents without gambling pathology [27]. After adjustment for psychiatric disorders and age, pathological gambling was associated with the report of at least one sleep complaint at an adjusted OR of 3.44 (95% CI 1.54–7.71), with all three sleep complaints concurrently at 3.45 (1.50–7.91), and with the individual complaints of difficulty initiating sleep (2.30; 1.07–4.95), difficulty maintaining sleep (4.60; 2.09–10.13), and early morning awakening (3.97; 1.86–8.48). Problem gambling carried more modest—though still significant—associations, with adjusted ORs of 1.79 (1.14–2.82) for any sleep complaint, 2.14 (1.17–3.93) for all three complaints, 1.96 (1.20–3.19) for difficulty initiating sleep, and 1.80 (1.10–2.94) for early morning awakening; the association with difficulty maintaining sleep did not reach significance [27]. The Australian National Social Survey, pooled across three waves and comprising 3760 adults, confirmed the population-level association. Problem gambling correlated positively with both insomnia and poor sleep quality, and remained an independent statistical predictor of insomnia in regression models adjusted for alcohol misuse [28]. A severity-graded relationship was also detectable in community samples not in treatment. In a cross-sectional study of 96 non-treatment-seeking gamblers, the mean Pittsburgh Sleep Quality Index score increased progressively from 3.35 in recreational gamblers to 5.30 in problem gamblers and to 5.44 in pathological gamblers, and the mean Epworth Sleepiness Scale score rose from 4.13 to 5.81 to 8.69 across the same three strata [29]. Stratified clinical comparison confirmed the direction of the association in younger adults. In a secondary analysis of 152 young adults aged 18–29 years stratified into healthy controls, an at-risk gambling subgroup, and a GD subgroup, the GD subgroup scored higher than controls on the insomnia items of the Hamilton Rating Scale for Depression, with the effect concentrated on middle and late insomnia. The same subgroup scored worse than both controls and at-risk gamblers on the Hamilton Anxiety Rating Scale sleep item, while Epworth Sleepiness Scale scores did not differ significantly across the three strata [30]. Data from treatment-seeking gamblers reinforced the same picture. In a cross-sectional study of 59 adults seeking outpatient treatment for problem gambling, the Problem Gambling Severity Index predicted Pittsburgh Sleep Quality Index scores (β = 0.18, t = 3.22, *p* < 0.01), and self-regulatory capacity mediated the association between problem gambling and both sleep difficulty (β = −0.45, t = −3.45, *p* < 0.001) and adverse sleep-related habits (β = −0.28, t = −3.76, *p* < 0.001) [31]. Collectively, the sleep evidence indicates a consistent pattern of excess insomnia symptoms, poorer subjective sleep quality, and increased subjective daytime sleepiness—with a severity gradient extending from recreational through problem to pathological gambling.

### 3.4. Neurological Disease

The neurological pole of the somatic phenotype rests on a smaller evidence base than the cardiovascular, metabolic, and sleep dimensions, but the available observations point to two related manifestations—an epidemiological clustering with seizures and epilepsy, and a clinical link between gambling behaviors and frontal lobe epilepsy. In the prospective Prevention and Etiology of Gambling Addiction Study, seizures and epilepsy emerged among the strongest non-psychiatric correlates of probable pathological gambling in the Maryland cohort of 1195 participants followed for up to four years [16]. The prospective design and the deliberate oversampling of gamblers strengthen the directional interpretation, although the absence of a defined seizure semiology in the parent dataset limited the etiological characterization of the events. This finding is consistent with the clinical observation that frontal lobe epilepsy can cluster with gambling behaviors. In a 2021 neurology clinic study of 174 adults with epilepsy and 65 with other neurological conditions, patients with frontal lobe epilepsy were the only subgroup whose gambling participation rate did not fall below that of the general population. Among the identified gamblers, the proportion who screened positive on the Lie/Bet questionnaire [23] was ten times the general-population rate; one third reported signs of escalation, and these patients were more likely to be receiving levetiracetam or brivaracetam than the other gamblers in the series. [32]. The clinical implication that this suggests—that the prefrontal substrate whose structural alterations are documented in pathological gamblers may share a common neuroanatomical vulnerability with the frontal seizure focus—remains a hypothesis rather than an established mechanism, since diffusion imaging alterations do not, by themselves, establish epileptogenic vulnerability. The neurological dimension of GD may therefore extend beyond the convulsive endpoint and points to a shared frontal substrate whose precise mechanism, including any iatrogenic contribution from antiseizure or dopaminergic agents prescribed in the broader epilepsy and movement disorder populations, awaits dedicated investigation [16,32]. However, several alternative interpretations should be considered when reading the seizure-and-epilepsy cluster observed in the Maryland cohort. The parent dataset did not report the seizure semiology, so an unclassified proportion of the events may reflect non-epileptic seizures of psychogenic or syncopal origin rather than epileptic events. Concurrent psychiatric comorbidity, use of antiseizure or dopaminergic medications for co-occurring conditions, sleep deprivation, alcohol withdrawal, and reverse causality (in which pre-existing epilepsy contributes to gambling behavior through behavioral disinhibition or through iatrogenic dopamine-receptor stimulation—a mechanism that the frontal lobe epilepsy series of Heaney & Baxendale [32] arguably illustrates, given the over-representation of levetiracetam and brivaracetam among the identified gamblers) cannot be excluded on the available evidence. The neurological pole of the somatic phenotype should therefore be regarded as an emerging and insufficiently characterized dimension, whose empirical base is currently thinner than that of the cardiovascular, metabolic, and sleep dimensions, and whose consolidation awaits replication in larger, well-characterized cohorts with defined seizure semiology and formal exclusion of iatrogenic and reverse-causality mechanisms.

## 4. Pathophysiology of the GD-Associated Somatic Phenotype

**Figure 1:**
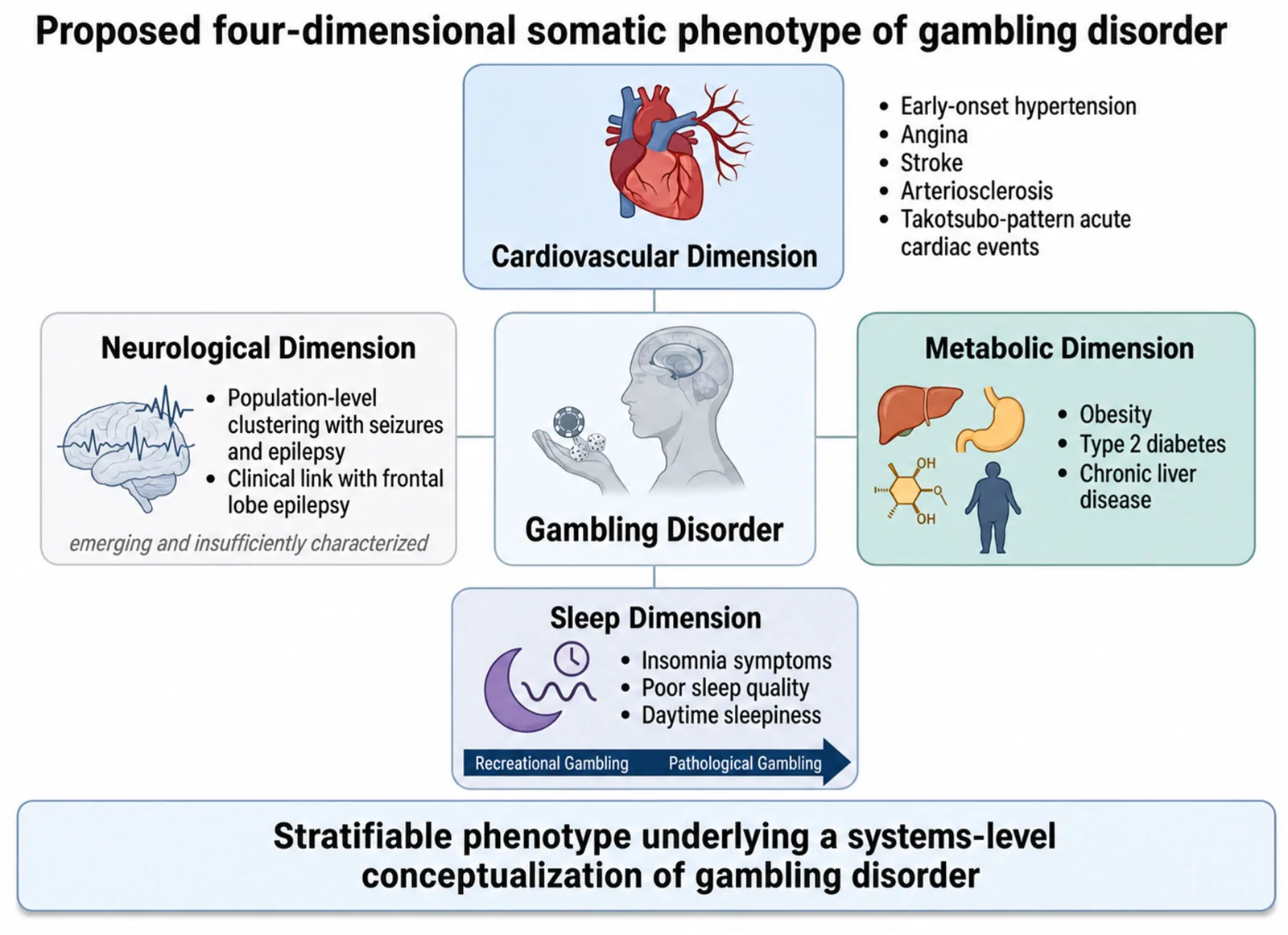
**Proposed four-dimensional somatic phenotype of gambling disorder**. Schematic representation of the cardiovascular, metabolic, sleep, and neurological dimensions that characterize the disorder at the population level. The four axes identify a stratifiable clinical phenotype upon which the systems-level conceptualization of gambling disorder can be built. The recreational-to-pathological gambling arrow is depicted under the sleep dimension, where the severity gradient is most explicitly documented in the empirical literature. The cardiovascular, metabolic, and sleep dimensions rest on multiple population-based estimates and, in several cases, on prospective or case–control designs, and are represented with full box opacity; the neurological dimension is currently supported by a smaller evidence base and is represented with reduced box opacity and the label “emerging and insufficiently characterized”.

**Figure 2:**
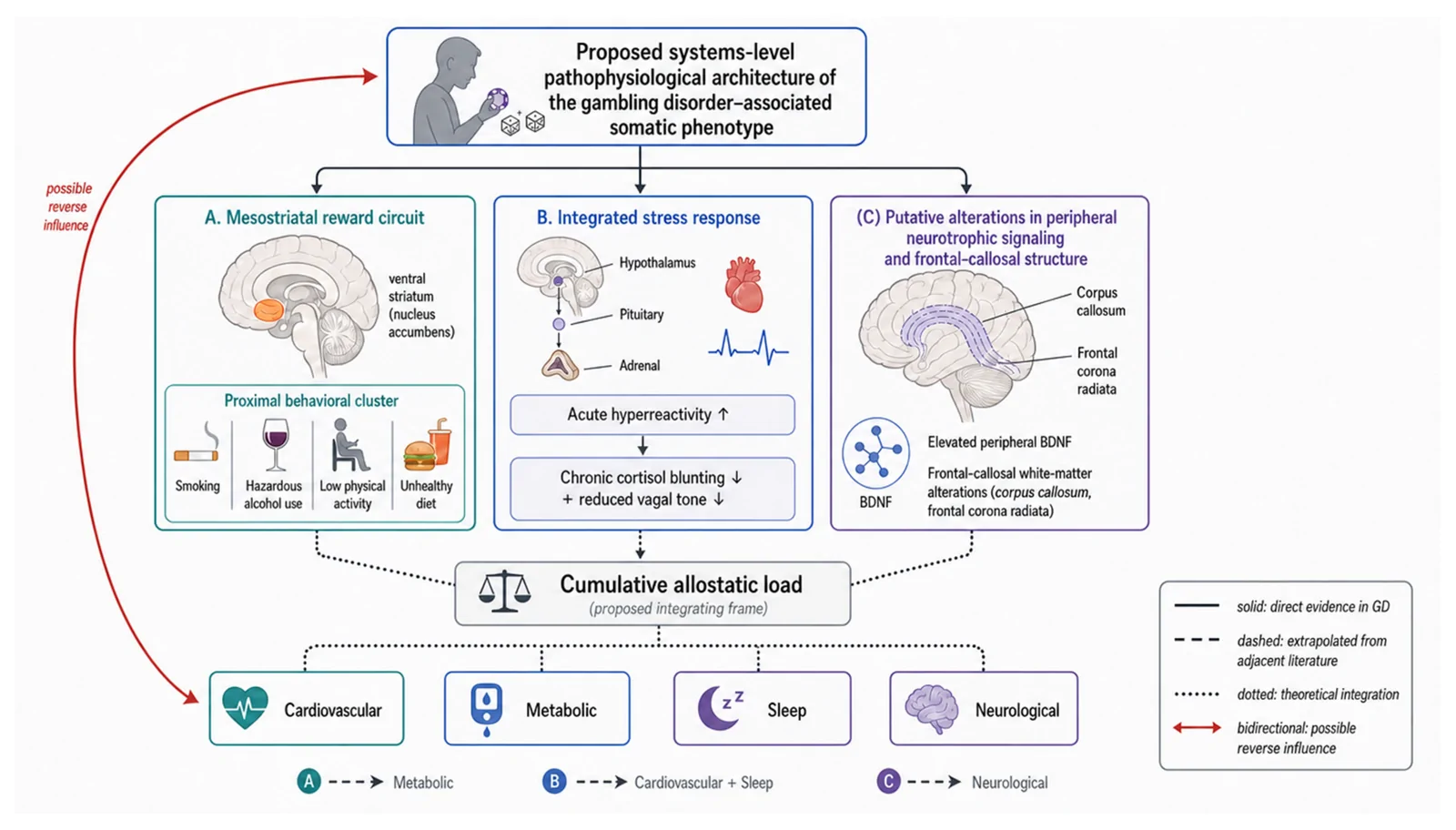
**Proposed systems-level pathophysiological architecture of the gambling disorder–associated somatic phenotype.** Repeated gambling exposure recruits three mechanistically coupled biological systems: (**A**) a hyperreactive mesostriatal reward circuit, anchored in the ventral striatum (nucleus accumbens), sustaining a proximal behavioral cluster of smoking, hazardous alcohol use, low physical activity, and unhealthy diet; (**B**) an integrated stress response transitioning from acute hyperreactivity to chronic basal cortisol blunting and reduced vagal tone; and (**C**) putative alterations in peripheral neurotrophic signaling and frontal-callosal structure, indexed by elevated peripheral brain-derived neurotrophic factor (BDNF) and by frontal-callosal white-matter alterations (corpus callosum, frontal corona radiata). The impaired inhibitory control imposed by axis C maintains the exposure (dashed arrow). Cumulative allostatic load is proposed as a conceptual integrating frame that may bring together the three axes and their convergence on the cardiovascular, metabolic, sleep, and neurological manifestations observed at the population level; this integration is hypothesized rather than directly demonstrated in individuals with GD. Line styles indicate the strength of evidence for each connection: solid lines mark pathways supported by direct evidence in individuals with GD (in particular the amphetamine-challenge PET evidence for mesostriatal hyperreactivity and its behavioral consequences); dashed lines mark pathways extrapolated from small mechanistic studies in GD combined with the broader literature in the general population (in particular the acute and chronic effects of the integrated stress response on the cardiometabolic and sleep phenotypes); dotted lines mark pathways proposed as theoretical integration and not directly demonstrated in individuals with GD (in particular the aggregation of the three biological axes through cumulative allostatic load and its convergence on the clinical outcomes). A bidirectional arrow between the clinical outcomes and the initial gambling exposure represents the possibility that chronic somatic illness, insomnia, disability, social isolation, financial stress, and psychological distress may themselves increase gambling behavior as a coping or emotion-regulation strategy.

The pathophysiological reading outlined here should be regarded as a model that integrates several converging lines of evidence, rather than as a demonstrated causal cascade. The somatic morbidity that characterizes GD (Figure 1) cannot be reduced to a parallel inventory of potential comorbid conditions. Within a systems-medicine perspective [33], each gambling episode can be conceptualized as a reward-driven behavioral event [34] whose physiological reverberations may propagate through three candidate biological systems—namely, the mesostriatal reward circuit with its proximal behavioral amplifier [35], the integrated stress response [9,36] that is proposed to translate the behavior into multi-system physiology, and the neuroplastic substrate [37] that may contribute to maintaining the exposure over time. Within this framework, the repetition transforms acute, adaptive responses into chronic dysregulated states whose reciprocal interactions converge on the cardiovascular, metabolic, sleep, and neurological manifestations observed at the epidemiological level. The architecture of this systems-level reading is illustrated schematically in Figure 2. The pathway represented here should be read as one direction of a bidirectional relationship: the reverse direction—from somatic and psychosocial burden to gambling behavior as a coping or emotion-regulation strategy—is a plausible complementary reading of the same associations.

The first axis is the mesostriatal reward circuit, which constitutes the upstream driver of the cascade. Positron emission tomography with the D2/D3 ligand [11C]-PHNO and an amphetamine challenge identified a 54–63% increase in dopamine release in the dorsal striatum of 12 pathological gamblers compared with controls [8], indicating a striatal hyperreactivity recruited by the salience cues of the gambling environment and driving the persistence of the behavior. The behavioral cascade that follows this neural priming is consistent across populations and clinically consequential. Heavy smoking, hazardous alcohol use, low physical activity, and an unhealthy diet have been reproducibly documented in patients with GD—with associations persisting after adjustment for sociodemographic and clinical covariates [22,24]. In addition, longitudinal data have documented the incident development of any substance use disorder within the natural history of GD (OR 2.61, *p* = 0.0036 for at-risk/problem/pathological gambling versus low-frequency/non-gambling at Wave 1) [38]. Each component of this cluster may carry independent pathogenic significance for the observed somatic associations. In this regard, nicotine produces sympatho-mimetic vasoconstriction and endothelial dysfunction, accelerating atherogenesis and adding a smoking-attributable fraction to the cardiovascular dimension [39]. Chronic alcohol consumption drives hepatic steatosis [40] and predisposes to liver fibrosis [41], accounting for the population-level association with liver diseases. Sedentary leisure and excess caloric intake produce insulin resistance and visceral adiposity [42] that interact with the chronic stress response associated with GD, potentially underpinning the associations with obesity and type 2 diabetes. However, the relative contribution of each component of the proximal cluster to the GD-attributable cardiometabolic burden has not been formally apportioned in the available datasets.

The second axis is the integrated stress response, which acts as the principal translational machinery between the behavior and the soma. Acute gambling engaged two stress-responsive systems at once—the sympathoadrenal axis and the hypothalamic–pituitary–adrenal axis. In a mechanistic study conducted under naturalistic casino conditions, blackjack play increased both heart rate and salivary cortisol concentrations [43]. Repeated activation transforms this acute response into a chronic profile of paradoxically blunted basal cortisol output—detectable in 35 patients tested with the Trier Social Stress Test, in whom basal cortisol correlated negatively with disease duration [36], and replicated in two further series at rest [44] and after amphetamine challenge, the latter accompanied by an altered baroreflex profile of elevated diastolic blood pressure and decreased heart rate over 90 min [45]. Parasympathetic regulation deteriorated in parallel; respiratory sinus arrhythmia reactivity was reduced in 22 patients compared with 22 matched controls, with the effect persisting after adjustment for age, smoking, and psychiatric comorbidities [46], and a partial replication was reported in a larger sample [47]. The downstream clinical consequences are multiple and reciprocally interconnected. Repeated sympatho-adrenergic surges directly raise arterial pressure and produce catecholamine-mediated endothelial injury [48]—mechanisms that may account for the cross-sectional excess of hypertension and angina observed in patients and for the takotsubo-pattern of acute cardiac decompensation reported in case series of high-stakes losses [20,21]. Diminished vagal output simultaneously withdraws the cholinergic anti-inflammatory brake on cytokine release [49], permitting the low-grade systemic inflammation that accelerates atherogenesis and may contribute to cerebrovascular events [50]. The same chronic activation disrupts circadian glucose homeostasis [51], raises cortisol concentrations [52], and promotes insulin resistance and central adiposity [53]—a second pathway, complementary to the behavioral one, that converges on the higher prevalence of type 2 diabetes and obesity observed in the disorder.

The altered sleep phenotype also functions as an amplifier that feeds back into the cardiometabolic axis. Experimental sleep restriction in healthy young men reduced glucose tolerance, raised evening cortisol concentrations, and increased sympathetic nervous system activity within a few nights [54], reproducing a neuroendocrine profile similar to that documented in pathological gamblers. The implication is that insomnia, poor subjective sleep quality, and daytime sleepiness observed in patients with GD are not merely consequences of the chronic arousal generated by the disorder but operate as potential independent drivers of further metabolic deterioration and cardiovascular risk. The result is a self-reinforcing loop in which gambling exposure produces hyperarousal and disrupts sleep, disrupted sleep amplifies cortisol and sympathetic tone the following day, and the resulting metabolic and vascular perturbations feed back into the cardiovascular dimension of the disorder.

The third axis concerns putative alterations in peripheral neurotrophic signaling and frontal-callosal structure that may contribute to maintaining the exposure over time. On the smaller neurological evidence base outlined above, four case–control studies have reported elevated serum brain-derived neurotrophic factor in pathological gamblers compared with controls, with replication across European and East Asian populations and a positive correlation with severity scores after adjustment for age, depression, and disease duration [44,55,56,57]. An additional within-patient study extended these findings to quantitative EEG correlations with cortical activation [58]. This peripheral signal has been interpreted as a putative compensatory upregulation in response to altered synaptic remodeling, with a heritable component suggested by polymorphisms in NTF3 and NTRK2 in a 357-participant case–control series [59]. Alternative explanations of the peripheral BDNF elevation should however be acknowledged: peripheral BDNF concentrations do not reliably index cerebral BDNF activity in humans, since BDNF is expressed and stored across multiple non-neural compartments (platelets, vascular endothelium, immune cells, adipose tissue), and its serum concentration can be modulated by age, sex, adiposity, physical activity, smoking, exercise habits, and concurrent psychiatric or inflammatory conditions—none of which was consistently controlled for across the four cited case–control studies [44,55,56,57]. The interpretation of the peripheral BDNF signal as evidence of altered cerebral neuroplasticity therefore remains a hypothesis rather than an established inference, and the signal may equally reflect a systemic, compensatory, or nonspecific response to the chronic behavioral and metabolic burden of the disorder. Three diffusion-weighted magnetic resonance imaging studies have reported reduced white-matter integrity in the corpus callosum and the frontal corona radiata of pathological gamblers [60,61,62], the most recent of which also documented reduced gray-matter volume in the left thalamus and reduced orbitofrontal thickness, with callosal integrity inversely correlated with gambling severity. The frontal-callosal substrate has been hypothesized to contribute to two mechanistically distinct effects within the systems-level architecture, neither of which has been directly demonstrated in individuals with GD. Specifically, it may impair the inhibitory control and decision-making that would otherwise interrupt the gambling exposure—with the potential consequence of preserving the chronic stress and behavioral cycles that may drive the cardiometabolic and sleep phenotypes—and it may plausibly predispose the cerebral cortex to anomalous excitability, a hypothesis compatible with the prospective clustering of the diagnosis with seizures and epilepsy at the population level and with the observation that frontal lobe epilepsy is the only neurological subgroup in which gambling participation does not fall below the general-population rate [16,32]. The alternative interpretations discussed at the closing of Section 3.4 (iatrogenic effects of antiseizure or dopaminergic medications, reverse causality, undocumented seizure semiology, non-epileptic seizures of psychogenic or syncopal origin) should however be borne in mind, and the causal architecture of these associations remains to be directly demonstrated.

Cumulative allostatic load has been proposed as a theoretical framework to integrate the multi-system observations described above [63]; to our knowledge, however, no study has quantified allostatic load in individuals with GD using a validated composite index—such as the ten-biomarker index of Seeman and colleagues or its subsequent multi-biomarker adaptations. The multisystem interpretation offered here therefore rests on the isolated physiological components assessed in the cited studies (cortisol, heart rate variability, blood pressure, respiratory sinus arrhythmia, behavioral risk factors), and the proposed integration through cumulative allostatic load is a hypothesis awaiting empirical testing in dedicated multi-biomarker cohorts that assess several physiological systems simultaneously in the same participants. Under this framework, the body mounts an adaptive multi-system response to each gambling episode, then progressively loses the capacity to return to baseline as the exposure is repeated and the behavioral cluster reinforces the physiological drive—with sleep disruption amplifying the daytime hormonal and autonomic disequilibrium and with the frontal-callosal substrate preventing the cessation of exposure that would allow recovery. The cardiovascular, metabolic, sleep, and neurological manifestations of the disorder are interpreted here as the hypothesized convergent end-points of this proposed multi-level process (Table 1).

## 5. Clinical Translation

The available evidence across epidemiology and pathophysiology can reshape the clinical approach to the patient with GD along three interrelated aspects, each carrying immediate operational implications for the medical contexts in which the disorder is encountered. Nonetheless, the clinical considerations offered in this section are proposed on the basis of the systems-level reading of the disorder rather than as an established standard of care, since the diagnostic yield, cost-effectiveness, and impact on clinical outcomes of a dedicated GD-specific medical screening pathway have not been directly evaluated.

The first area concerns the recognition of the clinical pattern that should prompt enquiry into a gambling history. When metabolic syndrome is present together with a substantial substance use and lifestyle risk burden and with chronic insomnia, the constellation identifies a presentation in which GD is a plausible upstream contributor—regardless of the order in which the somatic complaints come to medical attention. The pattern is not pathognomonic, and a single clinical enquiry will not establish the diagnosis on its own; the enquiry is, however, inexpensive and represents a natural entry point to the validated screening procedures and structured interviews on which a formal diagnosis is built [64].

Second, in patients with problem gambling or with a formal diagnosis of GD, the somatic evaluation may extend along the four phenotypic dimensions of the disorder. Within this evaluation, it is useful to distinguish three levels. The first level is general good clinical practice applicable to any adult (blood pressure measurement, fasting glucose or glycated hemoglobin, body mass index, tobacco and alcohol history, insomnia inquiry), which does not require a GD-specific rationale but is nevertheless often not systematically performed in patients presenting for behavioral or psychiatric care. The second level is targeted screening guided by conventional risk factors—a fasting lipid profile in patients above a given age or with cardiovascular risk factors, tests of hepatic function in patients with concurrent hazardous alcohol use or metabolic dysfunction, and polysomnographic assessment in patients whose anthropometric profile, witnessed apneas, or excessive daytime sleepiness raise the suspicion of obstructive sleep apnea. The third level is a hypothetically GD-specific evaluation: no direct evidence of incremental diagnostic yield or cost-effectiveness of a dedicated GD-specific screening pathway over conventional primary care applied to the same patient is currently available, and this third level is therefore proposed as a clinical consideration informed by the systems-level reading of the disorder rather than as an established standard of care. Under this framework, the cardiovascular evaluation would combine blood pressure documentation, fasting lipid profile, and global cardiovascular risk stratification; the metabolic evaluation would include anthropometric measures of BMI and abdominal circumference, glucose homeostasis by fasting glucose or glycated hemoglobin, and routine tests of hepatic function; the sleep evaluation would proceed through validated self-report instruments [65], with referral for polysomnographic study reserved for patients meeting the risk profile named above. The neurological evaluation would begin with a seizure history—which forms part of a comprehensive medical interview in any patient—and would proceed to formal neurological assessment only in patients with focal motor or behavioral symptoms, episodic disinhibition, or a suggestive family history that individually justify referral by conventional criteria [66]. Empirical evaluation of the diagnostic yield, cost-effectiveness, and clinical impact of a dedicated GD-specific screening pathway is a priority for future research.

The third aspect concerns the organization of care, and its implications may be considered bidirectionally. Gambling treatment services—traditionally constructed around psychological and psychiatric intervention [67]—should enlarge their first-line assessment pathway through the inclusion of a routine medical evaluation, with referral lines toward internal medicine activated whenever the assessment uncovers a clinically relevant abnormality. Internal medicine and its subspecialties, in turn, can acquire an upstream behavioral diagnosis to consider whenever the clinical presentation matches the pattern outlined above. We believe that this bidirectional flow should not be considered an accessory addition to existing pathways but an intrinsic property of the medical management of a disorder whose somatic phenotype emerges from the interaction between a behavioral exposure and a physiological response. Addressing one component of that interaction without the other would therefore leave the loop intact and the long-term trajectory potentially unchanged.

## 6. Potential Therapeutic Implications: GLP-1 Receptor Agonists

The therapeutic translation of the systems-level conception of GD outlined here requires an intervention capable of acting concurrently on the behavioral drive that maintains the exposure and on the cluster of somatic conditions that the exposure produces. Among the currently available drugs, GLP-1 RAs warrant exploratory consideration in this context, because the central activity of these drugs on reward-related circuits [68] and their peripheral cardiometabolic profile [69] intersect with the loci at which the disorder produces its multi-dimensional burden. In the absence of clinical or observational data in GD itself, the rationale developed below draws on mechanistic considerations and on phase 3 evidence in adjacent, non-gambling populations.

GLP-1 receptors are expressed in the ventral tegmental area and the nucleus accumbens—key nodes of the mesolimbic dopaminergic projection involved in addictive behaviors—and their activation suppresses phasic dopamine release in the nucleus accumbens, with downstream consequences for reward-driven behaviors [70]. The dopaminergic system in pathological gamblers shows altered responsivity at the level of the dorsal striatum, as documented by positron emission tomography during amphetamine challenge [8], pointing to a broader dysregulation of the dopaminergic projection on which GLP-1 RA action converges. In adjacent addictive conditions—in particular alcohol use disorder [71] and nicotine dependence [72]—GLP-1 RAs have attenuated craving and the heaviness of consumption episodes without affecting the frequency of use, a pharmacodynamic signature that points to selective modulation of the reward circuit rather than to a global behavioral suppression, and one that maps directly onto the cue-driven, episodic phenomenology of pathological gambling [73].

Beyond this neural foundation, the class carries a profile of somatic effects whose correspondence with the four phenotypic dimensions of GD is unusual in scope. Within the cardiovascular dimension, the SELECT trial—conducted in patients with established cardiovascular disease and with overweight or obesity in the absence of diabetes—documented a meaningful reduction in major adverse cardiovascular events under once-weekly subcutaneous semaglutide (hazard ratio 0.80, 95% CI 0.72–0.90) [74], in a population whose clinical profile substantially overlaps with that of the patient with GD seen for premature hypertension or for cardiovascular risk stratification.

Within the metabolic dimension, which throughout this review includes obesity, type 2 diabetes, and chronic liver disease as its three principal manifestations, the class has established benefits on body weight, glycemic control, and the broader trajectory of metabolic dysfunction [75]. Specifically on the hepatic component of this dimension, the ESSENCE trial has added direct histological evidence, with semaglutide producing resolution of steatohepatitis in 62.9% of treated patients compared with 34.3% on placebo, and reduction in liver fibrosis in 36.8% versus 22.4% [76]. Although the etiological profile of chronic liver disease in pathological gambling has yet to be characterized, the overlap between the metabolic features of the disorder and the indication of the trial—taken together with the documented behavioral risk cluster—may render this hepatic activity relevant to a substantial fraction of patients.

The extrapolation from GLP-1 RA effects on obstructive sleep apnea to the insomnia phenotype of GD requires particular caution. The SURMOUNT-OSA program demonstrated that tirzepatide reduced the apnea-hypopnea index by approximately 25–29 events per hour in adults with obesity and moderate-to-severe obstructive sleep apnea [77]. However, the sleep manifestations of GD are insomnia, poor subjective sleep quality, and daytime sleepiness, whose pathophysiology (arousal-driven, hypothalamic–pituitary–adrenal and sympatho-adrenal activation, circadian misalignment) differs substantially from that of obstructive sleep apnea (anatomical airway collapse compounded by adiposity). A substantial fraction of the SURMOUNT-OSA effect furthermore appears to be mediated by weight loss, a mediator that is unlikely to be operative in the majority of patients with GD, whose insomnia is not weight-dependent. The extrapolation of the SURMOUNT-OSA finding to the sleep phenotype of GD is therefore not warranted on the current evidence, and any effect of GLP-1 RAs on insomnia associated with GD remains an open question that would require dedicated evaluation.

The neurological extrapolation is even more preliminary. Two preclinical studies have reported that liraglutide reduces seizure kindling and prevents behavioral comorbidities in mouse models of pentylenetetrazole-induced and intrahippocampal kainic acid epileptogenesis [78,79]. These findings are hypothesis-generating for the neuroprotective properties of GLP-1 receptor activation in general, but they do not, in themselves, support a therapeutic role for the class in the neurological pole of the GD-associated phenotype: the models concern experimentally induced epilepsy in rodents rather than the frontal-callosal alterations and the epidemiological clustering with seizures documented in individuals with GD. The connection is currently too indirect to support any translational conclusion, and this part of the therapeutic rationale should be read as a preclinical mechanistic signal rather than as evidence for a GLP-1 RA effect on the neurological dimension of GD.

A dedicated randomized controlled trial of a long-acting GLP-1 RA in patients with GD would need to specify its target population. Two distinct designs are conceivable: (i) enrollment restricted to patients with GD who also carry an approved indication for the class (obesity, type 2 diabetes, or established cardiovascular disease with overweight), where the somatic benefit would be independently expected and the primary question would concern the additional effect on gambling severity and craving; (ii) enrollment of unselected patients with GD irrespective of concurrent metabolic or cardiovascular indications, where the primary question would concern the direct effect on gambling behavior in the absence of an approved somatic indication. The first design is more immediately feasible and clinically defensible; the second raises off-label prescribing questions that are addressed in the paragraph on adverse effects and accessibility below. In either design, composite endpoints should cover gambling severity scores such as the Problem Gambling Severity Index, craving measures, and parameters of the cardiovascular, metabolic, sleep, and neurological dimensions that define the phenotype. No clinical evidence currently supports the use of GLP-1 RAs in gambling disorder, and the candidate role of the class should therefore be regarded strictly as a mechanistically grounded hypothesis for which a dedicated randomized controlled trial would be required before any clinical inference.

In addition, several practical considerations should be borne in mind before contemplating the use of GLP-1 RAs in patients with GD who do not carry an approved indication for the class. Common adverse effects include gastrointestinal symptoms (nausea, vomiting, diarrhea, constipation), which are dose-dependent and lead to discontinuation in a substantial minority of patients across the pivotal metabolic and cardiovascular trials. Less frequent but clinically relevant events include acute pancreatitis, cholelithiasis, and, in the pediatric-obesity trials, a signal for suicidal ideation whose causal relationship to the drug remains uncertain. Contraindications include a personal or family history of medullary thyroid carcinoma and multiple endocrine neoplasia type 2, based on preclinical rodent findings whose transposition to humans is debated. Chronic administration is required to maintain both metabolic and cardiovascular benefit, and treatment discontinuation is associated with substantial weight regain within 12–24 months; long-term safety data beyond five years remain limited. The current cost of the class is high, and accessibility remains uneven both within and across healthcare systems, with substantial off-label demand driving supply shortages that affect patients with approved indications. Off-label prescribing to patients with GD in the absence of an approved somatic indication would raise clinical, ethical, and health-economic questions that go beyond the scope of this review, and would not be supported by the current evidence base. The candidate role of GLP-1 RAs in GD should therefore be evaluated within the framework of a formal randomized controlled trial rather than through off-label prescribing in routine clinical practice.

## 7. Limitations

The present review is subject to several caveats. First, the cross-sectional architecture of most of the available literature restricts inferences about temporal sequence and causation. With the exception of the Maryland cohort, which has supplied prospective cardiovascular and convulsive data [16], and the older-adult NESARC sample, which has supplied three-year longitudinal estimates of incident arteriosclerosis and heart disease [19], the associations reviewed rest on observational snapshots. A formal exclusion of reverse causation—in which an early-onset cardiometabolic burden might itself promote disordered gambling through affect-regulation or coping mechanisms—is therefore not available within the existing evidence base. The paradox reported in the older-adult NESARC sample—where recreational gamblers showed lower diagnoses of arteriosclerosis and cirrhosis than non-regular gamblers, while disordered gamblers showed higher diagnoses of angina and arthritis [15,80]—plausibly reflects a selection effect, with healthier older adults continuing social gambling, and is a reminder of the caution required when drawing causal conclusions at the other end of the spectrum.

A related consideration concerns the direction of the observed associations. The review is organized around a forward pathway from repeated gambling exposure to somatic morbidity, but a reverse pathway is equally plausible on the current evidence and should be explicitly acknowledged. Chronic somatic illness, insomnia, disability, social isolation, financial stress, and psychological distress may themselves increase gambling behavior as a coping or emotion-regulation strategy, and the cross-sectional associations reviewed here may therefore reflect this reverse direction in addition to, or instead of, the forward pathway proposed in the framework. Arbitration between the two directions requires prospective study designs in which baseline somatic health is assessed before the onset of disordered gambling, causal-mediation analyses on longitudinal cohorts in which gambling exposure and its proximal behavioral cluster are characterized at separate time points, and—where feasible—genetically informed designs such as Mendelian randomization. Until such designs become available for the somatic outcomes considered here, the framework offered in this review should be read as a bidirectional relationship rather than as a predominantly one-way pathway from gambling to somatic disease.

Second, the proximal behavioral cluster of heavy smoking, hazardous alcohol use, low physical activity, and unhealthy diet is itself an independent set of risk factors for the somatic outcomes under consideration. Cowlishaw and colleagues showed in the 2007 Adult Psychiatric Morbidity Survey of England that the physical-health effects of problem gambling were largely attenuated once socioeconomic factors and substance use were accounted for [81]. The directional model articulated in the pathophysiological section, however, posits that the mesostriatal hyperreactivity documented in pathological gamblers [8] drives this proximal cluster, which in turn produces a substantial fraction of the cardiometabolic burden—an architecture in which the attenuation observed in conventional regression analyses is the expected behavior of a mediator rather than evidence against the upstream association. The distinction between confounder and mediator therefore matters not only for statistical inference but also for the conceptual reading of the evidence, and formal causal-mediation analyses applied to longitudinal cohorts in which gambling and the behavioral cluster have been characterized at separate time points would consolidate this aspect of the framework.

Third, several mechanistic studies that inform the pathophysiological reading rest on small samples—the principal positron emission tomography evidence for mesostriatal hyperreactivity derives from twelve patients [8], the stress-axis and respiratory sinus arrhythmia investigations from samples of twenty-two to thirty-five [36,46], and the diffusion-weighted imaging studies on the frontal-callosal substrate from comparable cohort sizes [60,61,62]. Replication in larger, multi-site cohorts is needed before the inferences drawn from them can be considered established, with particular attention to the female subgroups that the current literature substantially underrepresents.

Fourth, heterogeneity of definitions, populations, and instruments compounds these issues. Diagnostic thresholds have shifted across editions of the classifications; source samples are geographically concentrated in high-income Western populations, with limited representation of low- and middle-income contexts in which the prevalence of gambling has risen markedly over the last decade; self-report instruments dominate the sleep and behavioral assessments, while administrative coding dominates the cardiovascular and hepatic outcomes, with cross-registry heterogeneity in coding practices adding a further source of measurement variability.

The evidence base assembled here is almost entirely derived from high-income Western populations—in particular from the U.S. NESARC and NCS-R datasets, from the Maryland PEGASUS cohort, from Northern and Western European studies conducted in Sweden, Denmark, Finland, the UK, and Italy, and from the Australian National Social Survey. Systematic data from East, South and Southeast Asia, from Sub-Saharan and Northern Africa, and from Latin America are essentially absent, even though these regions have witnessed a rapid expansion of gambling exposure over the last decade—driven by mobile sports betting in Sub-Saharan Africa, by the legalization of online gambling in several Latin American jurisdictions, and by the growth of the casino industry in East and Southeast Asia. The cardiovascular, metabolic, sleep, and neurological associations reviewed here should therefore not be assumed to transpose unchanged to non-Western populations, in which baseline anthropometric profiles—in particular the lower BMI threshold for cardiometabolic risk in East Asian populations—dietary patterns, alcohol consumption prevalence, and cultural determinants of gambling behavior differ substantially from the source cohorts. The mechanistic evidence is even less generalizable, since none of the neuroimaging or neuroendocrine studies included cohorts from Asia, Africa, or Latin America; and the therapeutic implications outlined for GLP-1 RAs rest on phase 3 trials whose enrollment was also concentrated in high-income Western populations. The generation of primary epidemiological, mechanistic, and therapeutic evidence in these under-represented regions is therefore a priority for the further development of the framework proposed here. The literature search was also restricted to English-language publications, which may have excluded relevant evidence published in Asian, African, or Latin American journals in local languages.

Finally, the therapeutic translation outlined for GLP-1 RAs rests on mechanistic plausibility, the recent evidence in adjacent addictive conditions, in particular alcohol use disorder and nicotine dependence, and phase 3 trials in overlapping but distinct populations [70]—none of which substitutes for a randomized controlled trial in GD itself. The preclinical evidence on epileptogenesis is, moreover, restricted to two animal models and to a single agonist (liraglutide) [78,79], with no human counterpart currently available. The candidate role of the class must therefore be regarded as a hypothesis to be tested rather than a recommendation to be implemented. Taken together, these caveats invite caution but do not displace the convergent message of the evidence assembled across populations, designs, and outcomes.

The argument developed throughout this review rests on four evidence tiers of unequal strength: (i) direct epidemiological association in GD, drawn from population-based surveys, cohort studies, and case–control designs; (ii) mechanistic plausibility from the small mechanistic studies in GD reviewed in the pathophysiological section; (iii) clinical extrapolation from adjacent addictive disorders and from phase 3 trials of GLP-1 receptor agonists in overlapping but non-gambling populations; and (iv) preclinical evidence from animal models of epileptogenesis. The therapeutic claims regarding GLP-1 receptor agonists draw predominantly on the third and fourth tiers, and should be interpreted with the corresponding caution; the epidemiological and pathophysiological sections rest more heavily on the first and second tiers.

## 8. Conclusions

The body of evidence reviewed here is compatible with the working hypothesis that GD may be productively considered as a behaviorally driven systemic medical condition, whose putative somatic phenotype appears stratifiable and mechanistically plausible on the current evidence, and may prove operationally consequential within a precision medicine framework should the framework be corroborated by dedicated empirical work. The phenotype distributes across the cardiovascular, metabolic, sleep, and neurological dimensions detailed above, with the sleep dimension carrying a severity gradient from recreational through problem to pathological gambling. The pathophysiological reading rests on three overlapping biological systems—mesostriatal reward hyperreactivity recruiting a proximal behavioral cluster, an integrated stress response shifting from acute hyperarousal to chronic basal cortisol blunting and reduced vagal tone, and putative alterations in peripheral neurotrophic signaling and frontal-callosal white-matter architecture, indexed by elevated peripheral BDNF and by diffusion-weighted MRI findings—with cumulative allostatic load proposed as a conceptual integrating frame that awaits direct empirical testing. Clinically, somatic evaluation across the four dimensions may be considered in patients with GD, along the three-tier framework outlined in Section 5, rather than being universally required; conversely, patients seen in non-psychiatric clinics for early-onset hypertension or metabolic syndrome, a substance use and lifestyle risk burden, and chronic insomnia may reasonably be asked about a gambling history. GLP-1 RAs—with central activity on the reward circuit and phase 3 evidence across adjacent, non-gambling somatic domains—constitute an exploratory therapeutic hypothesis whose formal evaluation within a dedicated randomized controlled trial would be required before any clinical inference could be drawn, as detailed in Section 6. A tentative operational consequence, should the framework proposed here be corroborated by further evidence, would be a progressive narrowing of the current gap between addiction psychiatry and internal medicine, with the somatic burden of GD entering the field of view of the latter while the management of the addiction itself would remain within the established structures of the former. Whether other behavioral addictions admit an analogous reading is an open question for the broader nosology.

## Figures and Tables

**Table 1:** Proposed three systems-level pathophysiological axes of gambling disorder and their hypothesized clinical correspondences.

Proposed Mechanistic Axis	Hypothesized Clinical Correspondence	Primary References in Gambling Disorder
Mesostriatal dopaminergic hyperreactivity with proximal behavioral amplification (smoking, alcohol, sedentary lifestyle, unhealthy diet)	Metabolic dimension (obesity, type 2 diabetes, hepatic steatosis and cirrhosis), with secondary amplification of cardiovascular and sleep risk via the behavioral cluster	[8,22,24,38]
Acute hyperreactivity with chronic blunting of the integrated stress response (HPA axis, sympatho-adrenal axis, parasympathetic tone)	Cardiovascular dimension (hypertension, angina, stroke, takotsubo-pattern decompensation) and sleep dimension (hyperarousal-mediated insomnia), with concurrent contribution to insulin resistance and central adiposity	[20,21,36,43,44,45,46]
Altered neurotrophic signaling with frontal-callosal structural substrate	Neurological dimension (clustering with seizures, frontal lobe vulnerability), with persistent decision-making impairment	[56,59,60,61,62]

The primary references listed in the third column comprise (i) population-based epidemiology in individuals with gambling disorder, (ii) small mechanistic studies in individuals with gambling disorder, and (iii) preclinical or adjacent-population extrapolation. Direct causal demonstration of the three axes in individuals with gambling disorder is not yet available; the corresponding four evidence tiers on which the review rests are detailed in Section 7 (Limitations).

## Data Availability

No new data were created or analyzed in this study.

## Abbreviations

The following abbreviations are used in this manuscript:

BDNF	Brain-derived neurotrophic factor
BMI	Body mass index
CI	Confidence interval
DSM-5	Diagnostic and Statistical Manual of Mental Disorders, 5th edition
EEG	Electroencephalography
GD	Gambling disorder
GLP-1 RA	Glucagon-like peptide-1 receptor agonist
HPA	Hypothalamic–pituitary–adrenal
ICD-11	International Classification of Diseases, 11th edition
NCS-R	National Comorbidity Survey-Replication
NESARC	National Epidemiologic Survey on Alcohol and Related Conditions
OR	Odds ratio
PEGASUS	Prevention and Etiology of Gambling Addiction Study in the U.S.
PET	Positron emission tomography

## References

[1] Grant J.E., Odlaug B.L., Chamberlain S.R. (2017). Gambling disorder, DSM-5 criteria and symptom severity. Compr. Psychiatry.

[2] Brand M., Rumpf H.J., Demetrovics Z., Müller A., Stark R., King D.L., Goudriaan A.E., Mann K., Trotzke P., Fineberg N.A. (2022). Which conditions should be considered as disorders in the International Classification of Diseases (ICD-11) designation of “other specified disorders due to addictive behaviors”?. J. Behav. Addict..

[3] Galeazzi G.M., Marchi M., Castagnini A.C. (2025). Psychiatric morbidity and gambling disorder: A systematic review and meta-analysis of population-based surveys. Eur. Psychiatry.

[4] Mohan G. (2025). Frequency of gambling, mental health, and financial stress among young adults. BMC Public Health.

[5] Floyd C.G., Connolly A.J., Tahk R.K., Stall L.M., Kraus S.W., Grubbs J.B. (2025). The role of social deficits in the link between social gambling motives and problem gambling. J. Gambl. Stud..

[6] Yau Y.H.C., Potenza M.N. (2015). Gambling disorder and other behavioral addictions: Recognition and treatment. Harv. Rev. Psychiatry.

[7] Petry N.M., Ginley M.K., Rash C.J. (2017). A systematic review of treatments for problem gambling. Psychol. Addict. Behav..

[8] Boileau I., Payer D., Chugani B., Lobo D.S.S., Houle S., Wilson A.A., Warsh J., Kish S.J., Zack M. (2014). In vivo evidence for greater amphetamine-induced dopamine release in pathological gambling: A positron emission tomography study with [^11^C]-(+)-PHNO. Mol. Psychiatry.

[9] Pangborn N., Froude A.M., Barkovich L., Brassard S., Balodis I.M. (2025). Acute stress response dysfunction in problem gambling (PG) and relationships with features of addiction. Psychoneuroendocrinology.

[10] Pfaltz M.C., Schnyder U. (2023). Allostatic load and allostatic overload: Preventive and clinical implications. Psychother. Psychosom..

[11] Kristensen J.H., Baravelli C.M., Leino T., Pallesen S., Griffiths M.D., Erevik E.K. (2024). Association between gambling disorder and suicide mortality: A comparative cohort study using Norwegian health registry data. Lancet Reg. Health Eur..

[12] Salonen A.H., Latvala T.A., Vuori M., Levola J., Castrén S., Grönroos T. (2025). Gambling disorder and increased psychiatric comorbidity: A Finnish register-based study. Nord. Alkohol Nark..

[13] Karlsson A., Balem M., Hansson H., Håkansson A. (2025). Suicide and mortality in individuals with gambling disorder and matched case controls—A Swedish nationwide register study. J. Gambl. Stud..

[14] Morasco B.J., Pietrzak R.H., Blanco C., Grant B.F., Hasin D., Petry N.M. (2006). Health problems and medical utilization associated with gambling disorders: Results from the National Epidemiologic Survey on Alcohol and Related Conditions. Psychosom. Med..

[15] Pietrzak R.H., Morasco B.J., Blanco C., Grant B.F., Petry N.M. (2007). Gambling level and psychiatric and medical disorders in older adults: Results from the National Epidemiologic Survey on Alcohol and Related Conditions. Am. J. Geriatr. Psychiatry.

[16] Schluterman N.H., Billioux V.G., Brown J.P., Al-Hadidi A., Tracy J.K. (2025). The relationship between gambling disorder, physical and mental health, and substance use in Maryland. J. Gambl. Stud..

[17] Abdul Rahim Y., Fernandez-Aranda F., Jimenez-Murcia S., Håkansson A. (2023). A nationwide case-control study on cardiovascular and respiratory-related disorders in patients with gambling disorder in Sweden. Public Health.

[18] Stevens H., Cooper L., Agroia H. (2025). How does gambling correlate with chronic health conditions?. Mo. Med..

[19] Pilver C.E., Potenza M.N. (2013). Increased incidence of cardiovascular conditions among older adults with pathological gambling features in a prospective study. J. Addict. Med..

[20] Wittstein I.S., Thiemann D.R., Lima J.A.C., Baughman K.L., Schulman S.P., Gerstenblith G., Wu K.C., Rade J.J., Bivalacqua T.J., Champion H.C. (2005). Neurohumoral features of myocardial stunning due to sudden emotional stress. N. Engl. J. Med..

[21] Sharkey S.W., Lesser J.R., Zenovich A.G., Maron M.S., Lindberg J., Longe T.F., Maron B.J. (2005). Acute and reversible cardiomyopathy provoked by stress in women from the United States. Circulation.

[22] Black D.W., Shaw M., McCormick B., Allen J. (2013). Pathological gambling: Relationship to obesity, self-reported chronic medical conditions, poor lifestyle choices, and impaired quality of life. Compr. Psychiatry.

[23] Johnson E.E., Hamer R., Nora R.M., Tan B., Eisenstein N., Engelhart C. (1997). The Lie/Bet Questionnaire for screening pathological gamblers. Psychol. Rep..

[24] Algren M.H., Ekholm O., Davidsen M., Larsen C.V., Juel K. (2015). Health behaviour and body mass index among problem gamblers: Results from a nationwide survey. J. Gambl. Stud..

[25] Grant J.E., Derbyshire K., Leppink E., Chamberlain S.R. (2015). Obesity and gambling: Neurocognitive and clinical associations. Acta Psychiatr. Scand..

[26] Leppink E.W., Fridberg D.J., Redden S.A., Grant J.E. (2016). The intersection of obesity and the longitudinal course of problem gambling. Psychiatry Res..

[27] Parhami I., Siani A., Rosenthal R.J., Fong T.W. (2013). Pathological gambling, problem gambling and sleep complaints: An analysis of the National Comorbidity Survey: Replication (NCS-R). J. Gambl. Stud..

[28] Thorne H.B., Rockloff M.J., Ferguson S.A., Vincent G.E., Browne M. (2021). Gambling problems are associated with alcohol misuse and insomnia: Results from a representative national telephone survey. Int. J. Environ. Res. Public Health.

[29] Parhami I., Siani A., Rosenthal R.J., Lin S., Collard M., Fong T.W. (2012). Sleep and gambling severity in a community sample of gamblers. J. Addict. Dis..

[30] Austin H.A., Chamberlain S.R., Grant J.E., Baldwin D.S. (2024). Sleep problems and gambling disorder: Cross-sectional relationships in a young cohort. J. Gambl. Stud..

[31] Loft M.H., Loo J.M. (2015). Understanding the mechanisms underlying gambling behaviour and sleep. J. Gambl. Stud..

[32] Heaney D., Baxendale S. (2021). Epilepsy & gambling: Risk factors for problem gambling behaviors in people with epilepsy. Epilepsy Behav..

[33] Stéphanou A., Fanchon E., Innominato P.F., Ballesta A. (2018). Systems Biology, Systems Medicine, Systems Pharmacology: The What and The Why. Acta Biotheor..

[34] Li G., Zhang S., Le T.M., Tang X., Li C.R. (2020). Neural responses to reward in a gambling task: Sex differences and individual variation in reward-driven impulsivity. Cereb. Cortex Commun..

[35] Sescousse G., Janssen L.K., Hashemi M.M., Timmer M.H., Geurts D.E., Ter Huurne N.P., Clark L., Cools R. (2016). Amplified striatal responses to near-miss outcomes in pathological gamblers. Neuropsychopharmacology.

[36] Maniaci G., Goudriaan A.E., Cannizzaro C., van Holst R.J. (2018). Impulsivity and stress response in pathological gamblers during the Trier Social Stress Test. J. Gambl. Stud..

[37] Olsen C.M. (2011). Natural rewards, neuroplasticity, and non-drug addictions. Neuropharmacology.

[38] Pilver C.E., Libby D.J., Hoff R.A., Potenza M.N. (2013). Problem gambling severity and the incidence of Axis I psychopathology among older adults in the general population. J. Psychiatr. Res..

[39] Münzel T., Crea F., Rajagopalan S., Lüscher T. (2025). Nicotine and the cardiovascular system: Unmasking a global public health threat. Eur. Heart J..

[40] Niezen S., Trivedi H.D., Mukamal K.J., Jiang Z.G. (2021). Associations between alcohol consumption and hepatic steatosis in the USA. Liver Int..

[41] Caputo F., Penitenti F., Bergonzoni B., Lungaro L., Costanzini A., Caio G., De Giorgio R., Ambrosio M.R., Zoli G., Testino G. (2024). Alcohol use disorders and liver fibrosis: An update. Minerva Med..

[42] Andersson D.P., Kerr A.G., Dahlman I., Rydén M., Arner P. (2023). Relationship between a sedentary lifestyle and adipose insulin resistance. Diabetes.

[43] Meyer G., Hauffa B.P., Schedlowski M., Pawlak C., Stadler M.A., Exton M.S. (2000). Casino gambling increases heart rate and salivary cortisol in regular gamblers. Biol. Psychiatry.

[44] Geisel O., Panneck P., Hellweg R., Wiedemann K., Müller C.A. (2014). Hypothalamic-pituitary-adrenal axis activity in patients with pathological gambling and internet use disorder. Psychiatry Res..

[45] Zack M., Boileau I., Payer D., Chugani B., Lobo D.S., Houle S., Wilson A.A., Warsh J., Kish S.J. (2015). Differential cardiovascular and hypothalamic pituitary response to amphetamine in male pathological gamblers versus healthy controls. J. Psychopharmacol..

[46] Moccia L., Quintigliano M., Janiri D., De Martin V., Rogier G., Sani G., Janiri L., Velotti P., Gallagher M.W., Di Nicola M. (2021). Heart rate variability and interoceptive accuracy predict impaired decision-making in gambling disorder. J. Behav. Addict..

[47] Kennedy D., Goshko C.B., Murch W.S., Limbrick-Oldfield E.H., Dunn B.D., Clark L. (2019). Interoception and respiratory sinus arrhythmia in gambling disorder. Psychophysiology.

[48] Quarti-Trevano F., Seravalle G., Grassi G. (2021). Clinical relevance of the sympathetic–vascular interactions in health and disease. Biomedicines.

[49] Borovikova L.V., Ivanova S., Zhang M., Yang H., Botchkina G.I., Watkins L.R., Wang H., Abumrad N., Eaton J.W., Tracey K.J. (2000). Vagus nerve stimulation attenuates the systemic inflammatory response to endotoxin. Nature.

[50] Mensah G.A., Arnold N., Prabhu S.D., Ridker P.M., Welty F.K. (2025). Inflammation and cardiovascular disease: 2025 ACC scientific statement: A report of the American College of Cardiology. J. Am. Coll. Cardiol..

[51] Zsombok A., Desmoulins L.D., Derbenev A.V. (2024). Sympathetic circuits regulating hepatic glucose metabolism: Where we stand. Physiol. Rev..

[52] Yamanaka Y., Motoshima H., Uchida K. (2019). Hypothalamic-pituitary-adrenal axis differentially responses to morning and evening psychological stress in healthy subjects. Neuropsychopharmacol. Rep..

[53] Xia S., Wu H., Ye J. (2025). Sympathetic nerve activity in obesity-associated insulin resistance. Acta Pharm. Sin. B.

[54] Spiegel K., Leproult R., Van Cauter E. (1999). Impact of sleep debt on metabolic and endocrine function. Lancet.

[55] Angelucci F., Martinotti G., Gelfo F., Righino E., Conte G., Caltagirone C., Bria P., Ricci V. (2013). Enhanced BDNF serum levels in patients with severe pathological gambling. Addict. Biol..

[56] Choi S.W., Shin Y.C., Mok J.Y., Kim D.J., Choi J.S., Hwang S.S. (2016). Serum BDNF levels in patients with gambling disorder are associated with the severity of gambling disorder and Iowa Gambling Task indices. J. Behav. Addict..

[57] Geisel O., Banas R., Hellweg R., Müller C.A. (2012). Altered serum levels of brain-derived neurotrophic factor in patients with pathological gambling. Eur. Addict. Res..

[58] Kim K.M., Choi S.W., Lee J., Kim J.W. (2018). EEG correlates associated with the severity of gambling disorder and serum BDNF levels in patients with gambling disorder. J. Behav. Addict..

[59] Solé-Morata N., Baenas I., Etxandi M., Granero R., Forcales S.V., Gené M., Barrot C., Gomez-Peña M., Mena-Moreno T., Vintró-Alcaraz C. (2022). The role of neurotrophin genes involved in the vulnerability to gambling disorder. Sci. Rep..

[60] Bellmunt-Gil A., Vorobyev V., Parkkola R., Lötjönen J., Joutsa J., Kaasinen V. (2024). Frontal white and gray matter abnormality in gambling disorder: A multimodal MRI study. J. Behav. Addict..

[61] Joutsa J., Saunavaara J., Parkkola R., Niemelä S., Kaasinen V. (2011). Extensive abnormality of brain white matter integrity in pathological gambling. Psychiatry Res..

[62] Yip S.W., Lacadie C., Xu J., Worhunsky P.D., Fulbright R.K., Constable R.T., Potenza M.N. (2013). Reduced genual corpus callosal white matter integrity in pathological gambling and its relationship to alcohol abuse or dependence. World J. Biol. Psychiatry.

[63] McEwen B.S. (2007). Physiology and neurobiology of stress and adaptation: Central role of the brain. Physiol. Rev..

[64] Molander O., Månsson V., Berman A.H., Grant J.E., Wennberg P. (2023). Assessing gambling disorder using semistructured interviews or self-report? Evaluation of the Structured Clinical Interview for Gambling Disorder among Swedish gamblers. Assessment.

[65] Morgan K., Kucharczyk E., Gregory P. (2011). Insomnia: Evidence-based approaches to assessment and management. Clin. Med..

[66] Alonso Vanegas M.A., Arrotta K., Davis K., Jobst B.C., Kotagal P., Poduri A., Valencia I. (2024). Frontal lobe epilepsy: Bermuda’s triangle. Epilepsy Curr..

[67] Seel C.J., Jones M., Christensen D.R., May R., Hoon A.E., Dymond S. (2024). Treatment of harmful gambling: A scoping review of United Kingdom-based intervention research. BMC Psychiatry.

[68] Badulescu S., Tabassum A., Le G.H., Wong S., Phan L., Gill H., Llach C.D., McIntyre R.S., Rosenblat J., Mansur R. (2024). Glucagon-like peptide 1 agonist and effects on reward behaviour: A systematic review. Physiol. Behav..

[69] Wang X., Qi M., Yang L., Yang L., Wang X., Zhang F., Cui Y., Wang D., Wang Y., Lv W. (2025). GLP-1 receptor agonists synergistic effects of metabolic reprogramming and cardioprotection. Front. Endocrinol..

[70] Lista S., Ballerio M., López-Ortiz S., Pinto-Fraga J., Pareja-Galeano H., Emanuele E., Martín-Hernández J., Santos-Lozano A. (2026). GLP-1 receptor agonists at the crossroads of obesity and addiction: A review of shared neurobiology and translational evidence. Pharmacol. Biochem. Behav..

[71] Petrie G.N., Mayo L.M. (2025). GLP-1 receptor agonists for the treatment of alcohol use disorder. J. Clin. Investig..

[72] Ahmed G.U., Zehra E., Rasheed S., Akhtar S.O., Raza A.A., Mujaddadi K., Samadi A. (2026). GLP-1 receptor agonists for smoking cessation: A narrative review of weight management potential. Ann. Med. Surg..

[73] García-Castro J., Cancela A., Cárdaba M.A.M. (2023). Neural cue-reactivity in pathological gambling as evidence for behavioral addiction: A systematic review. Curr. Psychol..

[74] Lincoff A.M., Brown-Frandsen K., Colhoun H.M., Deanfield J., Emerson S.S., Esbjørg S., Hardt-Lindberg S., Hovingh G.K., Kahn S.E., Kushner R.F. (2023). Semaglutide and cardiovascular outcomes in obesity without diabetes. N. Engl. J. Med..

[75] Westermeier F., Fisman E.Z. (2025). Glucagon like peptide-1 (GLP-1) agonists and cardiometabolic protection: Historical development and future challenges. Cardiovasc. Diabetol..

[76] Sanyal A.J., Newsome P.N., Kliers I., Ostergaard L.H., Long M.T., Kjaer M.S., Cusi K., Linder M., Okanoue T., Ratziu V. (2025). Phase 3 trial of semaglutide in metabolic dysfunction-associated steatohepatitis. N. Engl. J. Med..

[77] Malhotra A., Grunstein R.R., Fietze I., Weaver T.E., Redline S., Azarbarzin A., Sands S.A., Schwab R.J., Dunn J.P., Chakladar S. (2024). Tirzepatide for the treatment of obstructive sleep apnea and obesity. N. Engl. J. Med..

[78] de Souza A.G., Chaves Filho A.J.M., Souza Oliveira J.V., de Souza D.A.A., Lopes I.S., de Carvalho M.A.J., de Lima K.A., Florenco Sousa F.C., Mendes Vasconcelos S.M., Macedo D. (2018). Prevention of pentylenetetrazole-induced kindling and behavioral comorbidities in mice by levetiracetam combined with the GLP-1 agonist liraglutide: Involvement of brain antioxidant and BDNF upregulating properties. Biomed. Pharmacother..

[79] Citraro R., Iannone M., Leo A., De Caro C., Nesci V., Tallarico M., Abdalla K., Palma E., Arturi F., De Sarro G. (2019). Evaluation of the effects of liraglutide on the development of epilepsy and behavioural alterations in two animal models of epileptogenesis. Brain Res. Bull..

[80] Loo J.M., Kraus S.W., Potenza M.N. (2019). A systematic review of gambling-related findings from the National Epidemiologic Survey on Alcohol and Related Conditions. J. Behav. Addict..

[81] Cowlishaw S., Kessler D. (2016). Problem gambling in the UK: Implications for health, psychosocial adjustment and health care utilization. Eur. Addict. Res..

